# Une pneumopathie interstitielle diffuse révélant un syndrome des antisynthétases: à propos de 2 cas

**DOI:** 10.11604/pamj.2019.32.40.17903

**Published:** 2019-01-22

**Authors:** Nizar El Bouardi, Amina Alaoui, Meriem Haloua, Youssef Lamrani, Meryem Boubbou, Mustapha Maaroufi, Baderdine Alami

**Affiliations:** 1 Service de Radiologie, Centre Hospitalo-Universitaire Hassan II, Fès, Maroc; 2 Faculté de Médecine et de Pharmacie, Université Sidi Mohamed Ben Abdellah, Fès, Maroc

**Keywords:** Syndrome des antisynthétases, pneumopathie infiltrante diffuse, anti-Jo1, pronostic, Antisynthetases syndrome, diffuse infiltrative lung disease, anti-Jo-1, prognosis

## Abstract

Le syndrome des antisynthétases (SAS) est une myopathie inflammatoire fréquemment associée à une atteinte pulmonaire, surtout parenchymateuse (pneumopathie infiltrante diffuse). Les manifestations extrathoraciques associées à l'atteinte pulmonaire peuvent orienter le diagnostic: myalgies, polyarthralgies, syndrome de Raynaud, hyperkératose érythémateuse palmaire fissuraire et fièvre. Devant un tableau clinique et radiologique évocateur, la présence d'anticorps anti-ARNt synthétases permet de confirmer le diagnostic notamment les anti-Jo1. L'atteinte pulmonaire constitue un facteur pronostic majeur d'où l'indication une thérapie immunosuppressive intensive fondée sur la corticothérapie, les immunosuppresseurs ou l'association des deux. Une meilleure sensibilisation pour cette affection à révélation pulmonaire permettra d'adopter une prise en charge rapide et adéquate, et d'améliorer par conséquent le pronostic.

## Abstract (en)

Anti-synthetase syndrome (ASS) is an inflammatory myopathy commonly associated with pulmonary involvement, especially parenchymal (diffuse infiltrative pneumonitis). Extrathoracic manifestations associated with pulmonary involvement can give an indication to the diagnosis: myalgias, polyarthralgias, Raynaud's syndrome, erythematous palmar hyperkeratosis with fissures and fever. Given the suggestive clinical and radiological picture, the presence of aminoacyl-transfer RNA (tRNA) synthetase antibodies enables to confirm, in particular, Anti Jo-1 antibody activity. Pulmonary involvement is a major prognostic factor, hence the indication for intensive immunosuppressive therapy based on corticosteroids, immunosuppressive medications or the association among them. A better awareness about this disorder revealed by pulmonary manifestations could enable early and adequate management and to improve patient's prognosis.

## Introduction

Le syndrome des antisynthétases est une myopathie inflammatoire primaire rare qui est caractérisée par l'association d'une myosite, d'anomalies cutanées caractéristiques à type d'hyperkératose érythémateuse palmaire fissuraire des mains et de syndrome de Raynaud ainsi qu'une polyarthrite inflammatoire. L'atteinte pulmonaire y est fréquente et peut être révélée par une pneumopathie infiltrante diffuse (PID). Sur le plan biologique, cette affection se caractérise par la présence d'auto-anticorps antinucléaires spécifiques appelés « antisynthétases » (AAS) ciblant les enzymes intervenant dans la transcription de l'ARNt d'où le nom du syndrome. Parmi les AAS, l'anticorps anti-Jo-1 a été le premier découvert et le seul utilisé en routine. Nous décrivons à travers ce manuscrit l'observation de 02 patients admis pour exploration d'une pneumopathie infiltrante diffuse, et dont le bilan étiologique était en faveur d'un syndrome des antisynthétases anti Jo-1 positif. Nous discuterons par la suite les principaux aspects cliniques, radiologiques et pronostic de cette affection.

## Patient et observation

**Figure 1:**
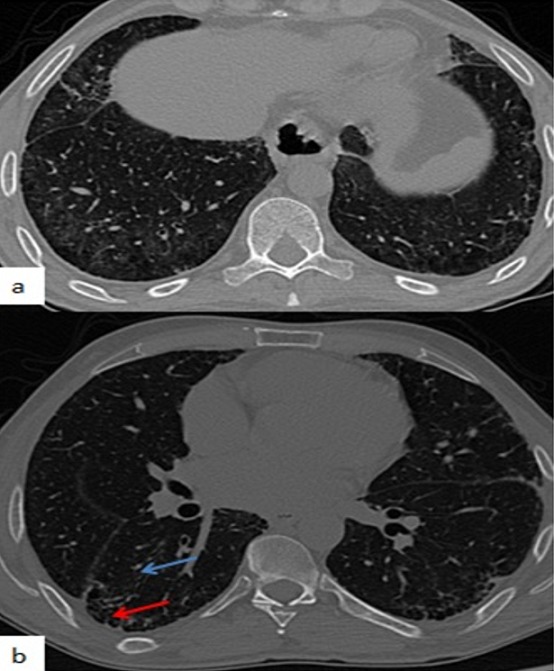
TDM thoracique en coupe axiale, fenêtrage parenchymateux(PINS): syndrome interstitiel pulmonaire fait de plages de verre dépoli, réticulations intra lobaires, d’images kystiques jointive à paroi épaisse (flèche rouge) (rayon de miel) ainsi que des bronchioléctasies par traction (flèche bleue) réalisant un aspect de PINS. Les anomalies prédominaient en postéro basal en bilatéral

**Observation 1**: il s'agit d'un patient de 37 ans, sans antécédents pathologiques notables, qui consulte pour une dyspnée lentement progressive sur une durée d'un an aggravée récemment devenue stade III de la NYHA. Cela s'associait à une polyarthralgie inflammatoire des petites articulations, de topographie bilatérale et symétrique, une hyperkératose érythémateuse palmaire bilatérale ainsi qu'une fièvre prolongée. L'examen clinique a objectivé des râles crépitants basithoraciques. L'examen de la marche de 6 minutes a dévoilé une désaturation de 95 à 85%. La spirométrie a mis en évidence un trouble ventilatoire restrictif. Devant ce tableau, une tomodensitométrie thoracique a été pratiquée et a révélé un syndrome interstitiel fait de plage de verre dépoli à prédominance lobaire inférieure, des réticulations intra lobulaire, des images kystiques jointives à paroi épaisses réalisant un aspect de rayon de miel ainsi que des bronchioléctasies par traction (Figure 1). Dans le cadre du bilan étiologique, le bilan biologique a permis d'écarter une vascularite en mettant en évidence des ANCA (anticorps anti cytoplasme des neutrophiles) négatifs, AAN (anticorps anti nucléaires) et Ac anti jo-1 positifs qui ont été demandés devant la forte présomption diagnostic d'un SAS. Les CPK étaient normales par ailleurs. Le diagnostic d'une pneumopathie infiltrative diffuse sur syndrome des antisynthétases a été retenu. L'électromyogramme n'avait pas noté d'atteinte musculaire. Le patient fut mis sous corticothérapie associée au cyclophosphamide avec amélioration partielle de sa dyspnée.

**Figure 2:**
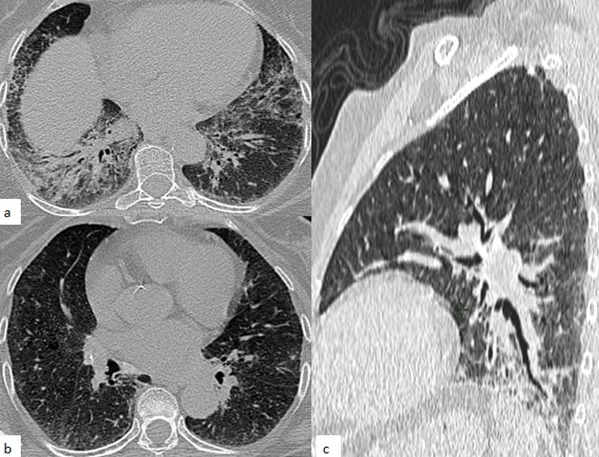
TDM thoracique en coupe axiale (a,b) et sagittale (c), fenêtrage parenchymateux: syndrome interstitiel fait de micronodules et nodules pulmonaires à distribution lymphatique centrolobulaire et sous pleurale, des foyers de verre dépoli bilatéraux au niveau des bases pulmonaires, un épaississement des septas interlobulaires, Ces anomalies sont à prédominance postéro basale et en bilatéral

**Observation 2**: patiente de 73 ans, diabétique type 2 sous antidiabétiques oraux, hypertendue, opérée pour carcinome vésiculaire de la thyroïde, qui consulte pour une dyspnée lentement évolutive (stade III de la NYHA), associée à une polyarthralgie de type inflammatoire. L'examen clinique objective des râles crépitants basithoraciques, ainsi qu'une hyperkératose érythémateuse fissuraire palmaire bilatérale réalisant un aspect de main de mécanicien. L'examen des masses musculaires a objectivé une discrète douleur exquise à la palpation. Devant ce tableau clinique, une tomodensitométrie thoracique a été réalisée objectivant un syndrome interstitiel fait de micronodules et nodules pulmonaire à distribution centrolobulaire et sous pleuraux, des foyers de verre dépoli bilatéraux au niveau des bases pulmonaires, un épaississement des septas interlobulaires, ainsi qu'un petit foyer de condensation lobaire inférieur gauche. Les lésions étaient à prédominance postéro basales (Figure 2). Le bilan biologique avait objectivé un syndrome inflammatoire biologique, AAN+, ANCA-, Anti-Jo-1 positif. Les CPK étaient élevées. L'électromyogramme montrait un tracé myogène. Le diagnostic d'une pneumopathie infiltrante diffuse sur syndrome des antisynthétases a été retenu. La patiente fut mise sous corticothérapie et cyclophosphamide avec amélioration partielle de sa dyspnée (stade II de la NYHA).

## Discussion

Les myopathies inflammatoires idiopathiques, définies en 1975 par Peter et Bohan [1] sont caractérisées par: une faiblesse musculaire proximale progressive, une augmentation des valeurs de la créatine kinase sérique, des anomalies caractéristiques à l'électromyographie et un infiltrat inflammatoire des muscles squelettiques à la biopsie. L'étude des autos anticorps associés à cette entité pathologique a permis de définir et classer différentes entités cliniques. Ainsi, le SAS a été décrit pour la première fois en 1990 par Margerie *et al*. comme une myosite inflammatoire primitive fréquemment associée à une PID et caractérisée par la présence d'auto-anticorps dirigée contre l'une des ARNt synthétases [2]. Le plus fréquents d'entre eux est l'anticorps anti-Jo1. Successivement, sept autres auto-anticorps ont été découverts (anti-PL12, anti-PL7, anti- OJ, anti-EJ, anti-KS, anti- Zo, anti-YRS). Cependant leur présence mutuelle est exceptionnelle. Le syndrome des antisynthétases constitue 30 à 35 % des myosites inflammatoires, son incidence reste rare rejoignant celle des myopathies inflammatoires évaluée actuellement à 11/100.000 habitants [3]. Il touche plus la femme que l'homme (sex ratio F/H:3:2), sans prédominance d'âge. L'atteinte pulmonaire au cours du SAS est retrouvée dans 67 à 100% [4]. Sa physiopathologie reste encore obscure, mais il semble que le poumon est l'organe où l'ARNt-synthétase devient anormalement immunogène. Cette atteinte s'associe plus fréquemment aux SAS à anti PL7, anti PL-12 et anti KS positifs [5]. En dehors des infections broncho-pulmonaires favorisées par l'atteinte de la musculature oropharyngée, les atteintes pulmonaires du SAS comprennent les atteintes parenchymateuses et les atteintes vasculaires. La PID a une prévalence de 77% [6]. Elle peut précéder le diagnostic du SAS (18%), être concomitante (64%), ou plus rarement apparaitre à posteriori [5]. Les symptômes respiratoires sont non spécifiques faits de toux et dyspnée. La fièvre peut s'y associer dans 26 % des cas [5]. Elle se présente sous 3 formes : PID aiguë, PID subaigüe /chronique et une PID asymptomatique de découverte fortuite sur un scanner thoracique systématique. Le scanner thoracique haute résolution reste l'examen clé pour l'exploration d'une PID associée à un SAS. L'acquisition spiralée est réalisée en fin d'inspiration profonde, patient en décubitus dorsal, avec des coupes natives fines (1-2 mm). La lecture se fait en double fenêtrage parenchymateux et médiastinal. L'usage des reconstructions multiplanaires (MPR) permet de déterminer le gradient apico basal et le mode maximum intensity projection (MIP) pour mieux détecter les micronodules. Les principales anomalies retrouvées par ordre de fréquence sont: l'atténuation en verre dépoli, les réticulations intra lobulaires, la condensation, le rayon de miel, les nodules et micro nodules pulmonaires, l'épaississement des septas interlobulaires et des lignes non septales, les bronchectasies par traction [7]. Ces anomalies prédominent en péri broncho vasculaire et postéro basale. Ces anomalies ont été présentes chez nos 2 patients avec comme pattern dominant le verre dépoli et les réticulations intra lobulaires. Les principales entités radiologiques sont par ordre décroissant: la pneumopathie interstitielle non spécifique (PINS), la pneumopathie interstitielle commune (PIC) et pneumopathie organisée cryptogénique (POC) [5, 7]. Il existe une bonne corrélation radio histologique, et de ce fait, la biopsie pulmonaire n'est plus obligatoire, de plus qu'elle n'est pas dénuée de risque de complications [7]. L'aspect histologique le plus retrouvé est celui de PINS, mais aussi de POC et PIC [8]. L'atteinte aiguë, correspondant au dommage alvéolaire aigue, peut mimer une pneumonie infectieuse avec cependant un bilan infectieux négatif et une mauvaise évolution sous antibiothérapie. Cette dernière est peu retrouvée vu que les patients les plus graves sont les moins biopsiés [8]. Les explorations fonctionnelles respiratoires (EFR) montrent classiquement un trouble ventillatoire restrictif avec une diminution de la diffusion locale du monoxyde de carbone (DLCO). Elles permettent certes l'orientation du diagnostic mais aussi l'évaluation de la gravité et le suivi des patients. Le syndrome restrictif peut être majoré par l'atteinte de la musculature respiratoire due à la myosite. Les résultats du lavage bronchiolo alvéolaire (LBA) sont très aspécifiques, montrant une alvéolite à CD8 ou une alvéolite neutrophilique associée à une éosinophilie [9]. Elle permet néanmoins d'écarter les diagnostiques différentiels notamment une mycobacteriose, une virose ou une pneumopathie d'hypersensibilité. L'hypertension artérielle pulmonaire (HTAP) est une complication vasculaire rare mais sévère au décours du SAS. Sa prévalence constitue 7,9% [10], avec un délai moyen de survenue de 7 ans par rapport au diagnostic du SAS selon une étude multicentrique menée par Hervier *et al*. portant sur 203 cas [10]. Cette prévalence rejoint celle de la survenue d'HTAP au cours des autres connectivites notamment le lupus et la sclérodermie [11]. A la différence des autres HTAP, celle du SAS est toujours associée à une PID et ne peut pas être isolée [10]. Le mécanisme de sa survenue se rapproche de celui des HTAP sur pathologie pulmonaire chronique entrainant une vasoconstriction hypoxique avec remodelage vasculaire secondaire. Cependant, plusieurs études ont démontré la discordance entre la sévérité de la PID et le développement d'une HTAP, et l'aggravation de l'HTAP malgré la stabilité de l'atteinte parenchymateuse pulmonaire [12]. Les signes cliniques évoquant une HTAP dans le cadre dans SAS sont une dyspnée disproportionnée par rapport à l'atteinte parenchymateuse pulmonaire et des signes d'insuffisance cardiaque droite. Comme pour toutes les connectivites, il serait recommandé de réaliser un dépistage systématique par échocardiographie. Le diagnostic de certitude n'est confirmé que par le cathétérisme cardiaque droite permettant d'éliminer une HTAP post capillaire et de confirmer son origine pré capillaire (Pression artérielle pulmonaire moyenne > 25 mm Hg, pression capillaire pulmonaire >15 mm Hg, débit cardiaque bas ou conservé) [13].

L'atteinte extra thoracique au décours du SAS est très hétérogène, elle comporte:

**Une atteinte musculaire**: l'atteinte musculaire a permis de classer le SAS parmi les myopathies inflammatoires. Sa prévalence est de 74% à 100% et peut être dans certains cas inférieure à celle de la PID. Elle s'observe plus dans les SAS à Ac anti-Jo1 positif (70%) [6]. L'aspect clinique est variable, elle peut être sévère (déficit musculaire proximal), pauci symptomatique (fatigabilité à l'effort, myalgies), asymptomatique, ou absente (amyopathique). Une douleur à la palpation des masses musculaires devrait être recherchée à l'examen clinique. Le dosage de la créatine phospho kinase (CPK) doit être largement prescrit. Il est souvent positif sauf pour les formes amyopathiques volontiers associées aux formes anti -PL 7 et anti- PL 12. L'électromyogramme (EMG) montre un tracé myogène. L'IRM permet de guider un éventuel prélèvement biopsique. Ces 02 derniers restent inutiles devant l'association d'une PID, de signes extra thoraciques et la positivité de l'un des anticorps antisynthétases.

**Atteinte articulaire**: l'atteinte articulaire varie entre 16 et 94% [9]. Les polyarthralgies inflammatoires sont les plus fréquentes et les moins graves, prédominant sur les petites articulations. L'arthrite érosive est beaucoup moins fréquente et plus grave. Les calcifications péri articulaires et les arthrites non déformantes ont été également décrites. La positivité du facteur rhumatoïde ou des anticorps anti peptides citrulinés définit une entité mixte de chevauchement avec la polyarthrite rhumatoïde [14].

**Atteinte cutanée**: **mains de mécanicien**: il s'agit d'une hyperkératose fissuraire et érythémateuse de la face latérale des doigts. Sa prévalence est de l'ordre de 16 à 21 %. Bien que rare et aspécifique, elle permet d'orienter le diagnostic [15].

**Phénomène de Raynaud**: sa prévalence est de l'ordre de 50%; de sévérité variable, pouvant être responsable dans les cas extrêmes d'ulcère digitaux [16].

Autres manifestations cutanées peuvent être présentes notamment les papules de Gottron, l'érythème lilacé des paupières, sclérodactylie ou télangiectasie.

**Autres atteintes plus rares**, **cardiaques**: la péricardite reste la plus fréquente et est associée à l'anticorps anti PL7.

**Digestives**: l'atteinte œsophagienne est la plus prédominante, suivie de l'atteinte oropharyngée, causales de fausses routes, dysphagie et de reflux gastro œsophagien rebelle au traitement [7]. Par ailleurs, une altération de l'état général et amaigrissement ont été rapportés. La fièvre peut être présente dans 87 % notamment dans les formes actives et PID aiguë [9]. La découverte d'une PID ou d'une fibrose pulmonaire incite à rechercher des signes clinico biologiques de connectivites et pratiquer un dosage des anticorps anti nucléaires, facteur rhumatoïde et anticorps anti peptides citrulinés. Ainsi, devant la positivité de l'anticorps antinucléaire, on procédera au dosage des anticorps anti synthétases devant un tableau fort évocateur du SAS. Néanmoins, il faudrait savoir demander la recherche spécifique d'anticorps anti-ARNt synthétases devant un tableau clinique compatible associant myalgies, hyperkératose palmaire, phénomène de Raynaud et/ou augmentation inexpliquée des CPK, même en l'absence d'anticorps antinucléaires. La méthode de référence est l'immunofluorescence indirecte sur cellules Hep2. La fluorescence est cytoplasmique car les anticorps anti ARN t synthétases font partie des anticorps anti antigènes nucléaires solubles et seront dirigés contre les protéines du cytosol. L'anticorps anti-JO1 est systématiquement recherché et est positif chez 2/3 des patients. Par ailleurs la recherche des autres anticorps anti ARN t synthétases doit être spécifiquement demandé car elle requiert des techniques plus spéciales [17]. L'atteinte pulmonaire interstitielle au cours du SAS est un élément de mauvais pronostic. Trois évolutions sous traitement ont été décrites : régression (20%), stabilité (80%), aggravation (20%) [18]. En effet, l'hétérogénéité des PID et les traitements immunosuppresseurs utilisés rendent le pronostic à long terme difficilement prévisible [17].

Néanmoins, quelques facteurs pronostiques ont pu être établis:

**Le mode de révélation**: la PID aiguë associant souvent dyspnée et fièvre est associée à un taux de mortalité élevé notamment si la dyspnée est sévère (stade III ou IV de la NYHA) [9]. L'atteinte des muscles respiratoires et la pneumopathie d'inhalation surajoutée semblent aggraver le pronostic.

**Le type histologique**: le dommage alvéolaire aigu est de pronostic sombre. La POC semble être de meilleur pronostic comparativement aux autres formes de PID notamment la PINS et la PIC [19].

**L'atteinte vasculaire**: à type d'HTAP est un facteur de mauvais pronostic. Cela justifie un suivi régulier et prolongé des patients atteint de SAS. Les éléments à surveiller comprennent les paramètres cliniques (évaluation de la faiblesse musculaire, dyspnée, crépitants, signes cutanés). Les investigations musculaires (enzymes+/- EMG), l'imagerie thoracique et EFR ainsi que le dépistage d'une HTAP par échocardiographie. Le taux d'AAS peut devenir indosable en cas de guérison [20].

La prise en charge du SAS doit être globale et axée l'atteinte pulmonaire. La corticothérapie systémique (prédnisone à dose de 1mk/kg/j) associée aux mesures adjuvantes (calcium, vitaminothérapie D, potassium, régime hyposodé, bisphosphonates) reste le traitement de référence [21]. Parfois inefficace seule, elle nécessite par ailleurs l'adjonction d'un immunosuppresseur dans plus d'1/3 des cas [22]. Les immunosupresseurs les plus utilisés sont le cyclophosphamide, l'azathioprine, le tarcolimus et le mycophénolates mofétil [22]. Une médication prophylactique à base de triméthoprime sulfametoxazol (400/80mg/j) est recommandée en cas d'association corticoïde et immunosuppresseur. La thérapie ciblée à base de rituximab (anti CD20) semble être prometteuse pour les cas réfractaires [23]. La perfusion d'immunoglobuline est indiquée en cas de trouble de déglutition ou de pneumopathie d'inhalation [24]. Les plasmaphérèses peuvent être indiquées dans les cas sévères mais avec une efficacité modérée [25]. La réhabilitation respiratoire et la prévention des infections pulmonaires par la vaccination anti grippale et anti pneumococcique sont toujours de mise. Il n'existe par ailleurs aucun traitement spécifique de l'HTAP au cours du SAS. Sa mise en route n'est pas recommandée du fait du risque d'aggravation du rapport ventilation perfusion et d'altération des échanges gazeux. Malgré toutes ces mesures thérapeutiques, la PID reste la principale cause de mortalité au cours du SAS. Elle la majore de 40% selon les études [26].

## Conclusion

Pour conclure, l'atteinte interstitielle pulmonaire reste très fréquente au cours du SAS. La présence de signes extra thoraciques caractéristiques et la positivité des anticorps anti ARN-t synthétases confirment le diagnostic. L'association corticoïdes et immunosuppresseurs se voit justifiée par la sévérité du pronostic et la mauvaise réponse à la corticothérapie seule chez ces patients. En plus des examens diagnostiques et de suivis classiques, les EFR apportent des éléments pertinents pour l'évaluation de l'efficacité thérapeutique. La recherche d'anticorps antisynthétases trouve actuellement son indication devant toute PID n'ayant pas fait preuve de son origine.

## Conflits d’intérêts

Les auteurs ne déclarent aucun conflit d´intérêts.
